# Unsupervised Clustering Reveals Distinct Subtypes of Biliary Atresia Based on Immune Cell Types and Gene Expression

**DOI:** 10.3389/fimmu.2021.720841

**Published:** 2021-09-27

**Authors:** Xiuqing Pang, Jing Cao, Shuru Chen, Zhiliang Gao, Guangjian Liu, Yutian Chong, Zhuanggui Chen, Jiao Gong, Xinhua Li

**Affiliations:** ^1^ Department of Infectious Diseases, Key Laboratory of Liver Disease of Guangdong Province, The Third Affiliated Hospital of Sun Yat-sen University, Guangzhou, China; ^2^ Guangzhou Women and Children’s Medical Center, Guangzhou Medical University, Guangzhou, China; ^3^ Department of Pediatrics, The Third Affiliated Hospital of Sun Yat-sen University, Guangzhou, China; ^4^ Department of Laboratory Medicine, The Third Affiliated Hospital of Sun Yat-sen University, Guangzhou, China

**Keywords:** autoimmune, perinatal infection, biliary atresia, immune cells and genes, unsupervised hierarchical clustering

## Abstract

**Background:**

Biliary atresia (BA) is a severe cholangiopathy of early infancy that destroys cholangiocytes, obstructs ductular pathways and if left untreated, culminates to liver cirrhosis. Mechanisms underlying the etiological heterogeneity remain elusive and few studies have attempted phenotyping BA. We applied machine learning to identify distinct subtypes of BA which correlate with the underlying pathogenesis.

**Methods:**

The BA microarray dataset GSE46995 was downloaded from the Gene Expression Omnibus (GEO) database. Unsupervised hierarchical cluster analysis was performed to identify BA subtypes. Then, functional enrichment analysis was applied and hub genes identified to explore molecular mechanisms associated with each subtype. An independent dataset GSE15235 was used for validation process.

**Results:**

Based on unsupervised cluster analysis, BA patients can be classified into three distinct subtypes: Autoimmune, Viral and Embryonic subtypes. Functional analysis of Subtype 1 correlated with Fc Gamma Receptor (FCGR) activation and hub gene *FCGR2A*, suggesting an autoimmune response targeting bile ducts. Subtype 2 was associated with immune receptor activity, cytokine receptor, signaling by interleukins, viral protein interaction, suggesting BA is associated with viral infection. Subtype 3 was associated with signaling and regulation of expression of Robo receptors and hub gene *ITGB2*, corresponding to embryonic BA. Moreover, Reactome pathway analysis showed Neutrophil degranulation pathway enrichment in all subtypes, suggesting it may result from an early insult that leads to biliary stasis.

**Conclusions:**

The classification of BA into different subtypes improves our current understanding of the underlying pathogenesis of BA and provides new insights for future studies.

## Introduction

Biliary atresia (BA) is a severe neonatal disease that often ends with cholestasis and progressive hepatic failure. Although BA is a rare disease, with variable incidence ranging from 1 in 5,000 to 1 in 19,000 live births per year, it is the leading cause of end-stage liver disease in children and the leading indication for pediatric liver transplantation worldwide (1). The development of the Kasai hepatic portoenterostomy (HPE) and liver transplantation have substantially improved patient outcomes. However, survival rates have been associated with the time-to-surgery and early diagnosis; even after successful HPE, progression to liver injury and fibrosis is still observed in 40%-50% of patients (1). After liver transplantation, the management of long-term complications and immune suppression is also a major clinical challenge (2, 3). Elucidating the pathogenesis underlying the extent and the source of the disease phenotype variance is key to improve current BA management and develop novel therapeutic strategies.

Pathological jaundice and acholic stools in the first few months of life are the clinical hallmarks of BA. Depending on time of disease onset, BA patients were initially divided into “embryonic/developmental” BA (<20%) and “perinatal/acquired” BA (>80%) (4). The presence of associated congenital anomalies such as polysplenia suggests a causative insult early in gestation and diagnosis of Embryonic BA is made. The absence of associated anomalies suggests that perinatal BA may be caused by a perinatal insult such as a viral infection. As of 2012, variants such as cyst-associated and cytomegalovirus(CMV)-associated BA have been added to the initial classification, based on presence of nonhepatic malformations, time of disease onset and morphological or molecular analysis of the hepatobiliary system (1, 4). Studies on the epidemiology, pathology and clinical forms of BA implicate numerous factors in disease pathogenesis, including: defective embryogenesis, abnormal fetal or prenatal circulation, genetic abnormalities, environmental toxins, viral infection, abnormal inflammation and autoimmunity, and susceptibility factors (1, 2). These seemingly different factors can be grouped into the broadly defined categories of abnormal morphogenesis, environmental factors and inflammatory dysregulation, which could interplay to produce a particular phenotype of BA. In this regard, analysis of gene expression patterns in BA can improve understanding of clinical heterogeneity and pathogenesis, determine disease prognosis, assist in clinical decision making, and may be the cornerstone of the design of personalized trials to block disease progression.

A growing body of evidence suggests the presence of activated immune cells in liver tissue of BA patients at the time of diagnosis, and an association with autoimmunity, amplification of epithelial injury, and bile duct obstruction (4–8). With substantial developments made in microarray technology and bioinformatics analysis, databases including Gene Expression Omnibus (GEO) have been increasingly used for enrichment analysis and identification of differential expressed genes (DEGs). Herein, we collected the BA microarray datasets from GEO, analyzed the immune-cell composition according to gene expression and tried to identify subtypes of BA at genetic and immune cell level. In this study, we applied a machine learning approach: unsupervised clustering on immune cells and gene expression to reveal the heterogeneity among BA patients and identified a *de novo* classification for BA. Then, validation was carried out using an independent BA dataset. Finally, we explored the mechanisms associated with each BA subtype, by identifying related immune cells and the hub genes.

## Materials and Methods

### Data Collection

Two datasets [GSE46995 (7) and GSE15235 (9)] were downloaded from the GEO database. GSE46995 used as a training dataset included 64 BA liver tissue samples, 14 diseased control samples (intrahepatic cholestasis) and 7 normal control samples, while GSE15235 consisting of 47 BA liver tissue samples was used for validation.

### Cluster Analysis

R package “CancerSubtypes” (10) was used for identifying, validating and visualizing molecular cancer subtypes from multi-omics data. Feature selection was based on the top 5000 most variance features. The ensemble Similarity Network Fusion and Consensus clustering algorithm (SNF-CC) was used to observe gene expression patterns across BA patients and cluster identification. Silhouette width index was calculated to assess accuracy and fitness of the clustering assignment. Silhouette width values range between -1 and 1. Values close to 1 correspond to well-defined clustering results. The xCell (11) function of “Immunedeconv”, another R package, was used for analyze infiltrating immune cells. xCell can show the relative enrichment of predetermined gene profiles combinations and perform cell type enrichment analysis from gene expression data for 64 immune and stroma cell types. The “Complexheatmap” package was then used for draw heatmaps.

### Differential Expression Analysis of Genes and Differences Among Different Subtypes

R package “tableone” was used to describe baseline characteristics. Comparison of genes between subtypes was performed using *t*-test and *p*<0.01 was used as the threshold value to test whether expression of DEGs was statistically significant. The representative DEGs were identified by a Venn plot method. For example, to identify the DEGs of subtype 1, a Venn plot of (subtype 1 + subtype 2) v/s (subtype 1 +subtype 3) was drawn to obtain the overlap of DEGs. *p*<0.05 represented statistically significant difference. Screening of immune and stroma cells for each subtype was performed by “tableone” and “*t*-test” and *p*<0.05 was used as the selection criteria. The screening method used to identify each BA subtype’s representative immune and stroma cells was the same as screening DEGs.

### Functional Enrichment Analysis and Protein-Protein Interaction Network Analysis

We performed enrichment analysis and Reactome pathway analysis using R packages “clusterProfiler” and “ReactomePA” to provide a functional interpretation of the representative genes. Then, the protein-protein interaction (PPI) network analysis was constructed by STRING (https://string-db.org/). The hub genes were analyzed, and the PPI network was visualized by cytoscape plug-in Molecular Complex Detection (MCODE) and CytoHubba of Cytoscape software version 3.7.2. MCODE was used to screen the modules of the PPI network identified by degree cutoff =2, node score cutoff =0.2, K core =2 and a maximum depth=100. Then, the importance of these hub genes was calculated by cytoscape plug-in CytoHubba, using the topological algorithm “degree”. In the PPI network, genes shown in red indicated more frequent interactions with other proteins.

## Results

### Subtype Identification of Biliary Atresia

**Figure 1:**
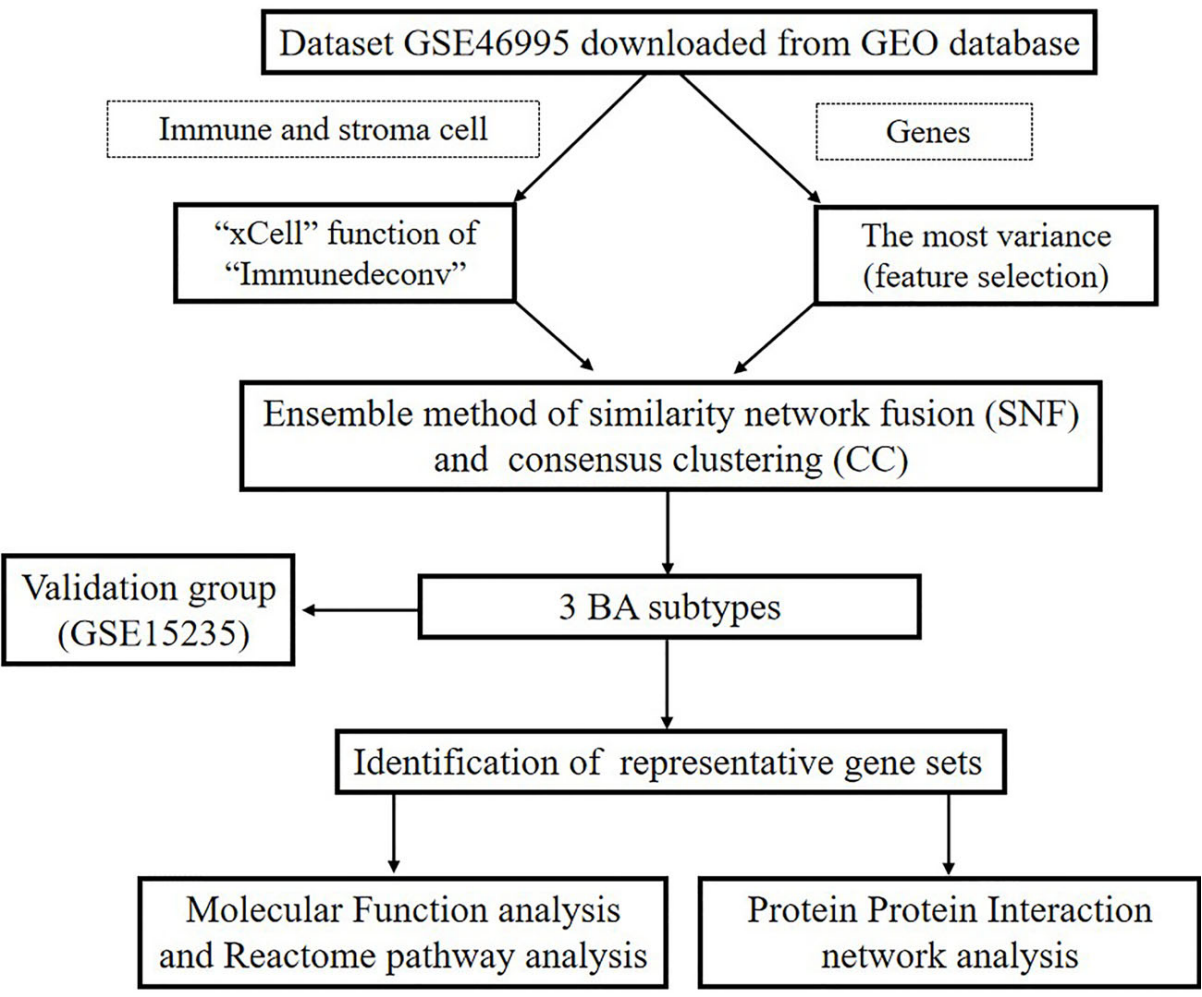
Flowchart of our research.

**Figure 2:**
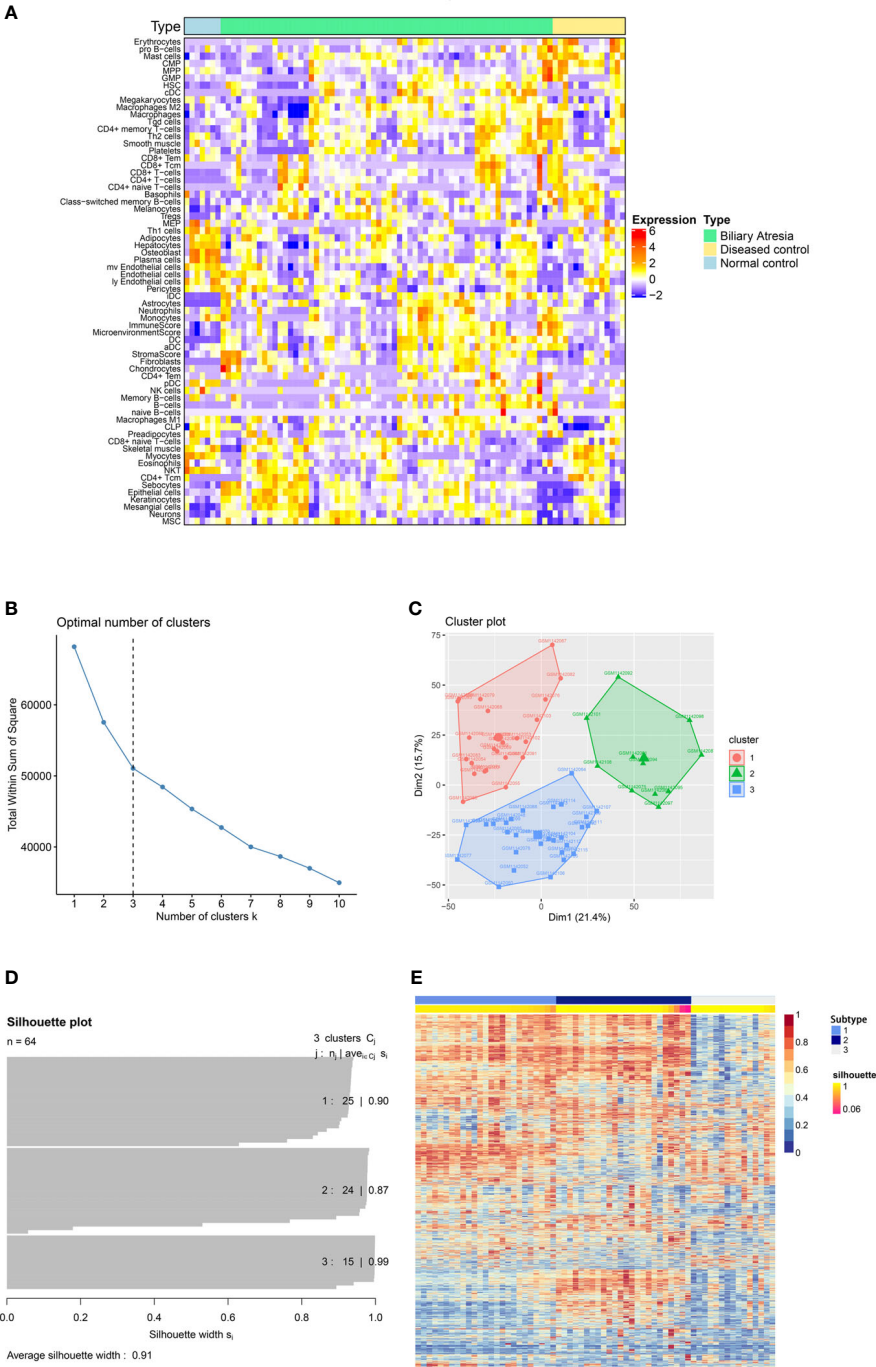
Identification of subtypes of Biliary atresia. **(A)** The immune and stroma cell composition based on analysis of dataset GSE46995. **(B)** The optimal number of clusters (K) was selected with factoextra package. **(C)** Visualization of cluster results using factoextra. **(D)** Silhouette width plots were drawn with CancerSubtypes package. **(E)** BA patients were clustered into three distinct subtypes by an ensemble method of SNF and CC (SNF-CC).

A flowchart of our study is illustrated in **Figure 1**. First, we downloaded GEO dataset GSE46995. Then we used xCell to assess the relative enrichment of gene profiles resulting in different immune and stroma cell types for each group (**Figure 2A**). We found significant differences in cell types among the three groups (**Supplementary Table 1**). In addition, the top 5000 most variance genes were selected by the most variance method. The optimal number of clusters (K) was generated according to “wss” (for total within sum of square) and visualized by factoextra package (K=3, **Figures 2B, C**). By applying an ensemble method of SNF-CC, BA datasets were clustered into three distinct subtypes (**Figures 2D, E**) with a silhouette width value of 0.91, suggesting a high degree of cohesion and separation among each subtype in the silhouette plot (**Figure 2D**). Validation was carried out using dataset GSE15235: 47 samples of BA were similarly clustered into three distinct subtypes based on gene expression and immune cells (**Supplementary Figure 1A**), with a silhouette width value of 0.89 (**Supplementary Figure 1B**). Gene Set Variation Analysis (GSVA) (12) was then applied to detect subtle pathway activity changes within the GSE46995 dataset, which similarly classified the samples into 3 subtypes (**Supplementary Figure 2**).

### Differential Expression Profile of BA Subtypes

**Figure 3:**
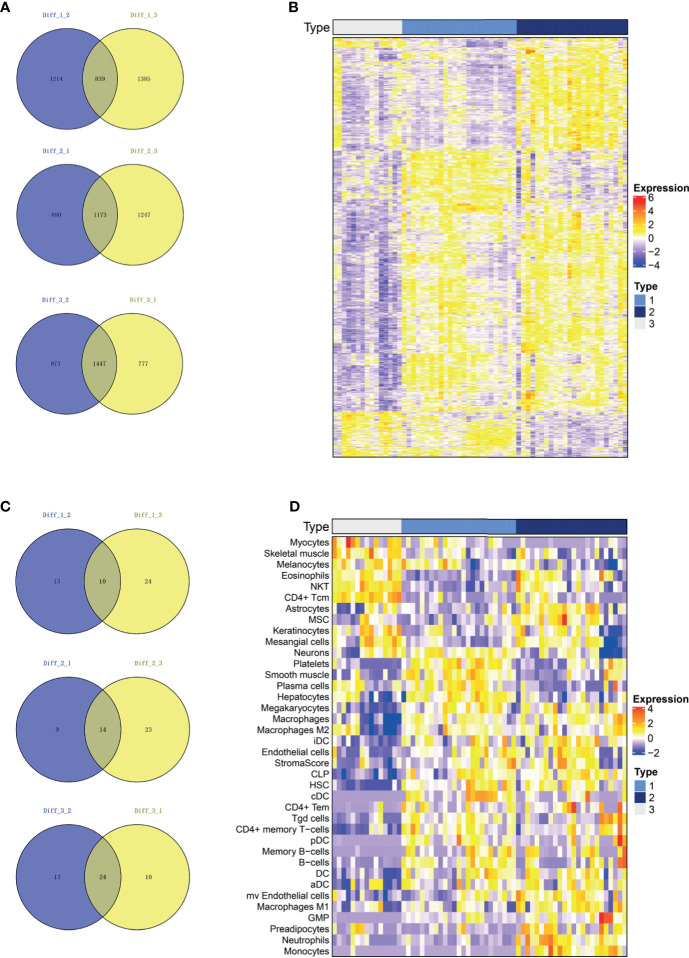
The representative genes and immune cells for each subtype. **(A)** The differential genes were calculated for each of the two subgroups and intersected. Subtype 1, Subtype 2 and Subtype 3 consisted of 839, 1173 and 1447 representative genes, respectively. **(B)** Heatmap of represented genes in all three subtypes. **(C)** We calculated the differential immune cells of the two subgroups and intersected them. Subtypes 1, 2 and 3 consisted of 10, 14 and 24 representative immune and stroma cells, respectively. **(D)** Heatmap of represented immune and stroma cells in all three subtypes.

A Venn plot was used to identify representative genes for each subtype (See method section 2.4 for more details). Subtypes 1, 2 and 3 consisted of 839, 1173 and 1447 representative differentially expressed genes, respectively (**Figure 3A**). The expression of these representative genes in three subtypes was then illustrated on a heatmap (**Figure 3B**). In addition, subtypes 1, 2, and 3 consisted of 10, 14 and 24 representative cells, respectively (**Figure 3C**), with the representative cells illustrated on a heatmap (**Figure 3D**). The representative cells in subtype 1 included platelets, smooth muscle, plasma cells, hepatocytes, macrophages, Hepatic Stellate Cells (HSC), B cells and Conventional dendritic cells (cDC). Subtype 2 included astrocytes, mesenchymal stem cells (MSC), iDC, aDC, microvascular endothelial cells, macrophages, macrophages M1, Granulocyte-Monocyte Progenitor (GMP), preadipocytes, neutrophils, monocytes and high stroma score while Subtype 3 included myocytes, skeletal muscle, melanocytes, eosinophils, Natural killer T cell (NKT) and CD4+ Tcm.

### Function and Pathway Enrichment Analyses

**Figure 4:**
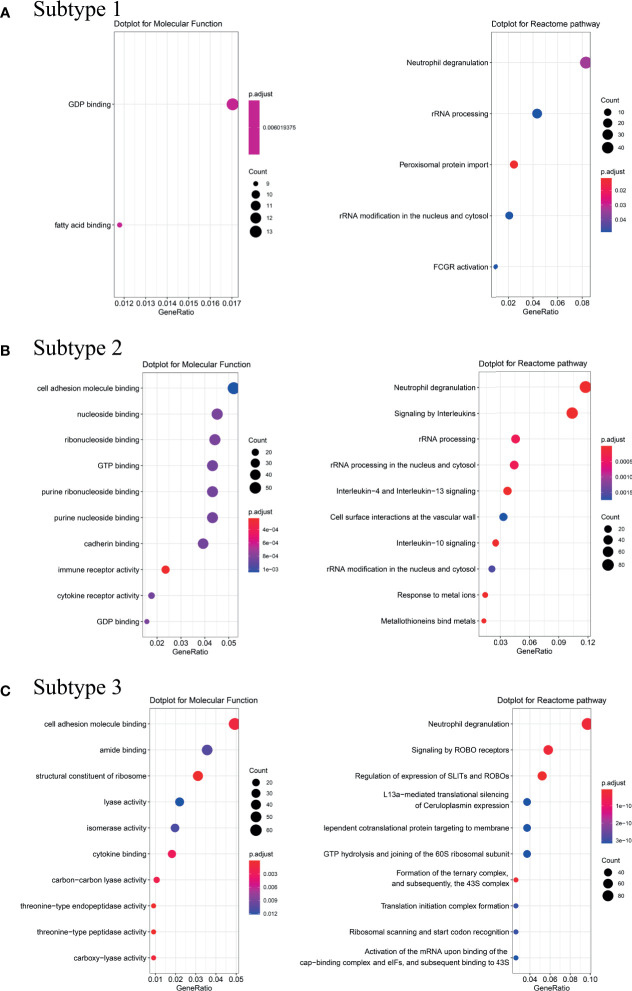
Analysis of Molecular Function and Reactome pathway. Molecular Function enrichment and Reactome pathway analysis were performed on the representative genes of the Subtype 1 **(A)**, Subtype 2 **(B)** and Subtype 3 **(C)**. Counts refer to the number of genes enriched into the relevant pathway and the larger the dotplot, the more genes are enriched. Different colors represented different *p*.adjust.

To explore possible pathological mechanisms in each BA subtype, we extracted the representative genes and performed Molecular Function (MF) enrichment and Reactome pathway analysis. MF analysis of genes from Subtype 1 showed correlation with GDP binding and fatty acid binding, while reactome analysis showed enrichment in neutrophil degranulation, rRNA processing and Fc Gamma Receptor (FCGR or FcγR) activation pathways (**Figure 4A**). MF analysis of the genes from Subtype 2 showed correlation with cell adhesion molecule and cytokine receptor activity, while Reactome analysis showed enrichment in neutrophil degranulation, signaling by interleukin such as IL-4, IL-13, IL-10, and response to metal ions pathways (**Figure 4B**). MF analysis of genes from Subtype 3 showed correlation with cell adhesion molecule binding and structural constituent of ribosome cytokine binding. Reactome analysis showed enrichment in neutrophil degranulation, signaling and regulation of expression of Roundabout (Robo) receptors pathways (see **Figure 4C**).

### Hub Genes of Different Subtypes

**Figure 5:**
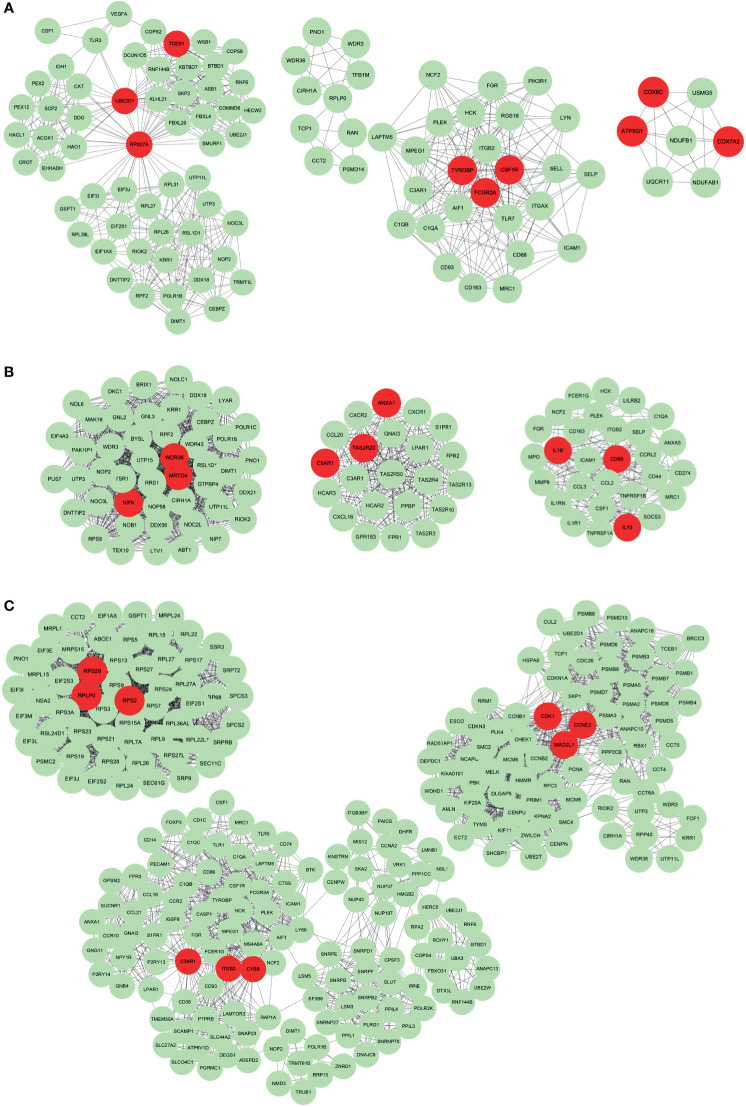
The protein-protein interaction network of representative genes of each subtype. The Protein-protein interaction network constructed by STRING was imported into Cytoscape for further analysis using the MCODE plug-in. The top 3 clusters from representative genes of Subtype 1 **(A)**, Subtype 2 **(B)** and Subtype 3 **(C)** are illustrated and related hub genes (red color) in each clusters were obtained from degree values.

We further identified hub genes from representative genes of each subtype and PPI networks were constructed (**Supplementary Table 2**), with results imported into Cytoscape for further module analysis. The MCODE plug-in was used to calculate the top 3 clusters, and degree values obtained hub genes in each cluster. The hub genes for Subtype 1, 2 and 3 are illustrated in **Figures 5A–C**, respectively. The hub genes for Subtype 1 included *TCEB1*, *UBE2D1*, *RPS27A*, *TYROBP*, *FCGR2A*, *CSF1R*, *COX6C*, *ATP5G1* and *COX7A2*. The hub genes for subtype 2 were *WDR36*, *MRTO4*, *NIFK*, *ANXA1*, *C5AR1*, *TAS2R20*, *IL1B*, *CD68* and *IL10*. Besides, the hub genes for subtype 3 were *RPS29*, *RPLP0*, *RPS2*, *C3AR1*, *ITGB2*, *CYBB*, *CDK1*, *CCNE2* and *MAD2L1*. The relationship between these pathways and representative genes was also visualized in **Supplementary Figure 3**. In Subtype1, *RPS27A* and *UBE2D1* were connection points between the peroxisomal protein import pathway and rRNA processing pathway (**Supplementary Figure 3A**). *FCGR2A* was a connection between the FCGR activation pathway and the neutrophil degranulation pathway (**Supplementary Figure 3A**). In Subtype 2, hub genes *IL-10*, *IL-1B* and *ANXA1* were enriched in pathways including signaling by interleukin such as IL-4, IL-13 and IL-10 (**Supplementary Figure 3B**). In Subtype3, hub genes *RPS29*, *RPLP0* and *RPS2* were at the junction of multiple pathways such as signaling and regulation of expression of Robo receptors, SRP-dependent co-translational protein targeting membrane translation initiation complex formation, etc. (**Supplementary Figure 3C**).

Since the “neutrophil degranulation” pathway was present in all 3 BA subtypes by Reactome Analysis, the genetic information associated with neutrophil degranulation was downloaded from the website: https://reactome.org/. The intersection of the above genes and representative genes in the three subtypes resulted in 35 overlapping genes. The expression of these 35 genes was significantly different in all BA subtypes (**Supplementary Table 3**). The differential expression of these 35 genes in BA and non-BA group was also analyzed (**Supplementary Figure 4**). 5 hub genes *TYROBP, FCGR2A, CD68, C3AR1* and *ITGB2* were then included. The differential expression of these five hub genes in the healthy subject group and the three BA subtypes was analyzed using R package “ggpubr”. As shown in **Supplementary Figure 5**, except for Subtype 3, the expression of hub genes *CD68*, *TYROBP, FCGR2A*, *C3AR1* and *ITGB2* was significantly different between the healthy group and Subtypes 1 and 2.

## Discussion

Herein, we provide new insights into the mechanisms underlying etiological heterogeneity and increase our understanding on the role of immune system in BA. By applying gene expression microarray and immune cell composition analysis, BA can be classified into three subtypes: Autoimmune, Virus-related and Embryonic subtypes.

Subtype 1 was associated with tissue cells including HSC, B cell, cDC, macrophages, platelets and hepatocytes. The transformation and proliferation of HSCs from fat-storing cells to myofibroblasts in BA have been reported to play an important role in hepatic fibrogenesis and BA pathogenesis, leading to increased extracellular matrix and progressive scarring (13). Immune tolerance in the liver is mediated by specialized antigen-presenting cells (APCs), including both HSC and DC, amongst others (14). Antigen-specific B cells responsible for initiation the humoral response play a major role in BA immunopathogenesis (4). B cells can act as APCs that on their own, activate T cells and the Th-1 pathway (15). Amy G. Feldman et al. found that B cells played a major role in the development of biliary obstruction in a Rhesus rotavirus (RRV)-induced mouse model of BA (15). cDCs are also essential for T cell activation and can present native antigens to B cells (16). In addition, reactome analysis showed enrichment in FcγR activation and hub gene Fc Fragment Of IgG Receptor IIa (*FCGR2A)* pathways. *FCGR2A*, formerly known as CD32, plays an important role in the clearance of immune complexes by phagocytic cells such as macrophages and neutrophils. Prior studies have demonstrated strong evidence that *FCGR2A* is not only involved in immune system activities but also with autoimmunity (17, 18). Based on the above findings, we can infer subtype 1 may be related to an autoimmune response targeting bile ducts which result into BA.

Interestingly, functional analysis of the representative genes of subtype 2 showed correlation between the DEGs and immune receptor activity, cytokine receptor activity and cell adhesion molecule binding by MF analysis. KEGG pathway analysis revealed significant viral protein interaction with cytokines and cytokine receptor (**Supplementary Figure 6**). Based on the above findings, we can infer that Subtype 2 may be related to infection and cytokine activity. Furthermore, the distinct immune cells in Subtype 2 included innate immune cells such as macrophages M1, neutrophils and monocytes. Intriguingly, CMV employs M1-associated markers and chemokines to promote the proinflammatory activation of infected monocytes/macrophages (19). CMV-induced pathologies are known to be mediated predominately by infected monocytes, which serve as a permissive system and are a long-term reservoir for the virus (20). In our study, CD68 was identified as the hub gene for Subtype 2. De facto, CD68 is the most extensively used marker, expressed on all macrophages (21). H Kobayashi et al. found macrophage-associated antigens (CD68) were associated with poor prognosis in BA patients (22). While much emphasis has been placed on CMV, other viruses such as the Epstein-Barr virus, reovirus and rotavirus have previously been documented in BA development. Since no clinical data such as CMV titer was available, we could only infer that Subtype 2 was related to perinatal viral infection. Moreover, the strong correlation with signaling by interleukin including IL-4, IL-13 and IL-10 was also found. This raises the possibility that the modulation of interleukin signaling may potentially represent a novel therapeutic approach to block progression of the disease in this particular subtype.

Reactome analysis of Subtype 3 showed enrichment within neutrophil degranulation, signaling and regulation of expression of Robo receptors pathways. The Robo family encodes transmembrane receptors that regulate axonal guidance and cell migration. Robo4 is an endothelial-specific receptor that participates in endothelial cell migration, proliferation, and angiogenesis as well as the maintenance of vasculature homeostasis (23). Hub gene Cyclin dependent kinase 1 (CDK1) has been primarily identified as a key cell cycle regulator in both mitosis and meiosis (24), and CCNE2 (cyclin E2) negatively regulates CCNE1 activity and S-phase progression during liver regeneration (25). A correlation between ITGB2 (CD18 protein) polymorphism and BA pathogenesis was substantiated by a prior study, suggesting the role of genetic predisposition in this particular subtype (26). Therefore, it can be inferred that subtype 3 may be related to embryonic BA as developmental defects of the liver and biliary tract are caused by genetic influences (1).

Interestingly, Reactome pathway analysis showed enrichment within the neutrophil degranulation pathway in all BA subtypes. Nowadays, much controversy surrounds the role of neutrophils in BA. The wide distribution of neutrophils in BA was previously documented by S. Changho (27). Intriguingly, further studies documented release of neutrophils following bile duct ligation (28, 29), suggesting that bile duct obstruction, whether due to edema induced by a viral infection or to other mechanisms, is the initial triggering mechanism that causes chemotaxis of neutrophils. Interestingly, the expression of hub genes (*C3AR1* and *ITGB2*) in subtype 3 was not significantly different from the healthy group (**Supplementary Figure 5**). This may be due to other representative genes that may play a more important role in neutrophil degranulation in this particular subgroup. A histological approach was initially adopted by Moyer et al. (9) to classify 47 BA patients into three groups: inflammation, fibrosis and unclassified groups. However, after undergoing molecular profiling, 4 cases were still left unclassified. Another focus of the study was to correlate each group with prognosis, for example the fibrosis group was associated with a significantly lower transplant-free survival than the inflammation group. However, the authors conceded that temporal differences in age at diagnosis for the molecular groups raise the possibility that the two gene expression signatures reflect two distinct but interrelated stages of the disease. Indeed, elements of inflammation and fibrosis are often visible simultaneously at BA diagnosis. Inflammatory signatures may be prominent in early stage of disease and can transition to fibrosis at more advanced disease stages. Herein, a different methodology was adopted since we aimed to explore etiological heterogeneity in BA by analyzing genetic signatures and characteristics of the microenvironment in BA.

One significant limitation of our study is our inability to correlate each BA subtype with prognosis. No survival data was available from the downloaded dataset. In our study, a high stroma score was obtained for Subtype 2 (viral-related). Clinical studies have consistently shown poorer prognosis of CMV positive patients after HPE (30, 31). Interestingly, worse survival rates have also been associated with tumors with high stroma scores (32).

Nonetheless, hospitals performing more than five cases per year had better surgical outcomes compared with hospitals performing a smaller number of HPE (33). At four years, significant differences in overall survival were found when HPE was done at a center with a higher caseload (34). Such reports suggest the difficulty in obtaining accurate prognosis data, especially when it comes to rare diseases. In addition, the use of xCell to characterize the tumor microenvironment has several limitations. Correlations with direct measurements are not perfect, and inferences being enrichment scores cannot be interpreted as proportions. Thus, xCell results should be interpreted with caution and require validation *via* other methods. Accordingly, our attempt to classify BA by integrating multi-omics data requires further validation *via in vivo* and *in vitro* studies.

## Data Availability Statement

The datasets presented in this study can be found in online repositories. The names of the repository/repositories and accession number(s) can be found in the article/**Supplementary Material**.

## Author Contributions

ZC, JG, and XL designed this study and took responsibility for the integrity of the data and the accuracy of the data analysis. XP and JC analyzed the data and wrote the article, SC, ZG, GL and YC contributed to revise the article. All authors contributed to data interpretation, and reviewed and approved the final version.

## Funding

This work was supported by a grant from the National Key R&D Program of China (2018YFC1315400), Guangdong Key Field R&D Plan (2019B020228001) and 5010 Project of Sun Yat-sen University (No. 2018024).

## Conflict of Interest

The authors declare that the research was conducted in the absence of any commercial or financial relationships that could be construed as a potential conflict of interest.

## Publisher’s Note

All claims expressed in this article are solely those of the authors and do not necessarily represent those of their affiliated organizations, or those of the publisher, the editors and the reviewers. Any product that may be evaluated in this article, or claim that may be made by its manufacturer, is not guaranteed or endorsed by the publisher.

## Abbreviations

APCs, antigen-presenting cells; BA, Biliary atresia; cDC, Conventional dendritic cell; CCNE2, cyclin E2; CDK1, Cyclin dependent kinase 1; CMV, cytomegalovirus; DEGs, differential expression genes; FCGR, Fc Gamma Receptor; FCGR2A, Fc Fragment Of IgG Receptor IIa; GEO, Gene Expression Omnibus; GMP, Granulocyte-Monocyte Progenitor; GSVA, Gene Set Variation Analysis; HPE, Kasai hepatic portoenterostomy; HSC, Hepatic Stellate Cell; PPI, protein-protein interaction; MCODE, Molecular Complex Detection; MF, Molecular Function; MSC, mesenchymal stem cell; NKT, Natural killer T cell; Robo, Roundabout; RRV, Rhesus rotavirus; SNF-CC, Similarity Network Fusion and Consensus clustering.
