# Supplementary material for: Unsupervised Clustering Reveals Distinct Subtypes of Biliary Atresia Based on Immune Cell Types and Gene Expression
Source: Front Immunol. 2021 Sep 27;12:720841. doi: 10.3389/fimmu.2021.720841 (PMC8502897; doi:10.3389/fimmu.2021.720841)
Supplement: Supplementary file 1 [file DataSheet_1.docx]

**Supplementary Figure 1.** Validation was performed using unsupervised cluster analysis on dataset GSE15235. (A) Heatmap displaying the differential expressed genes, immune and stroma cell composition of all 3 BA subtypes. (B) Silhouette plots for each identified BA subtype.


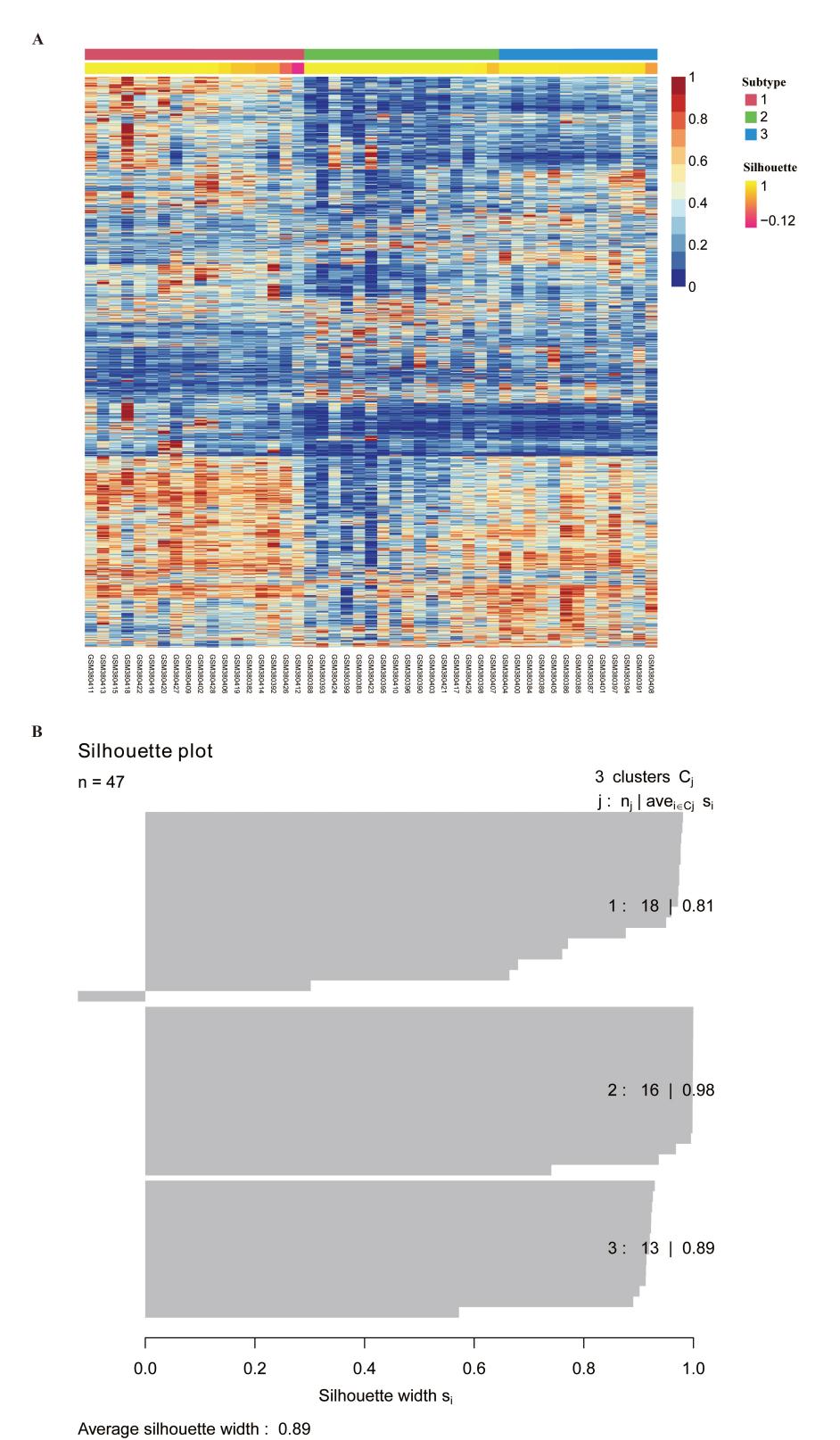


**Supplementary Figure 2.** Gene Set Variation Analysis (GSVA) performed on dataset GSE46995.

**Supplementary Figure 3.** The intricate relationship between hub genes and enriched Reactome pathways in each subgroup was visualized by “clusterprofile”. Panels A, B and C represents cnetplots for subtype 1, subtype 2 and subtype 3, respectively.


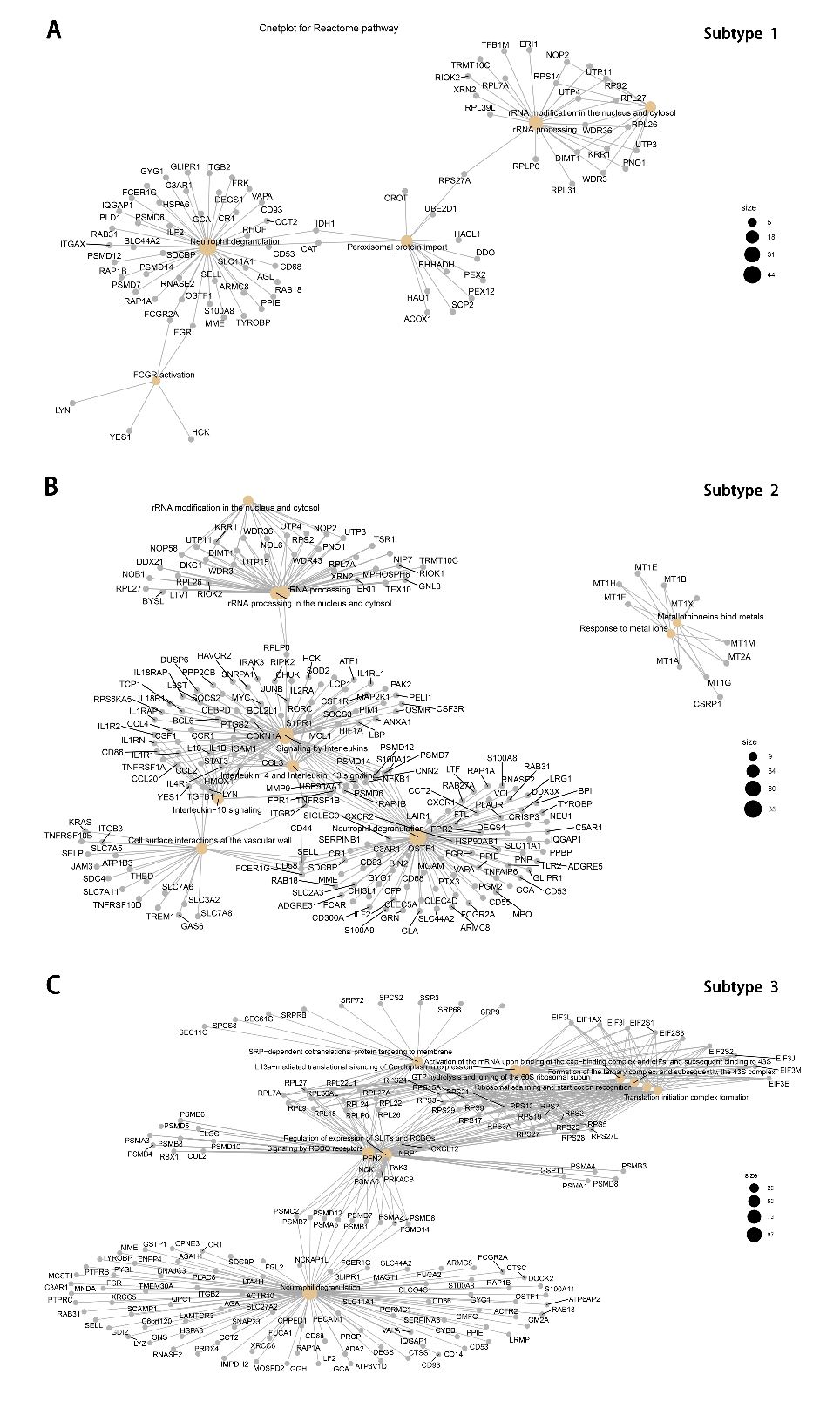


**Supplementary Figure 4.** The expression of 35 overlapping genes was related to neutrophil degranulation in the BA group and non-BA group. (*p*<0.05 indicated statistically significant difference)


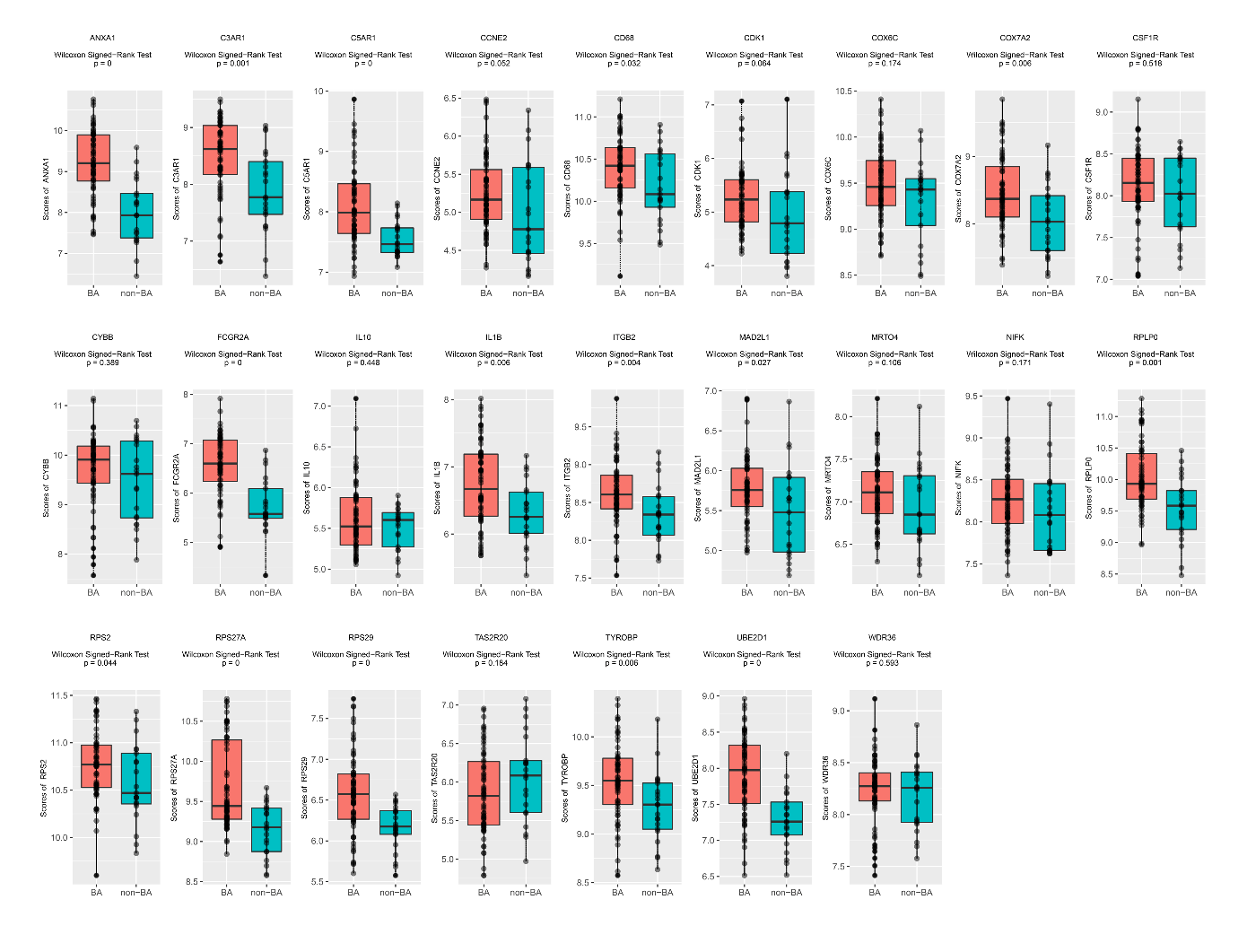


**Supplementary Figure 5.** Differential expression analysis of hub genes *TYROBP, FCGR2A, CD68, C3AR1* and *ITGB2* was performed in the healthy subject group and the three BA subtypes.


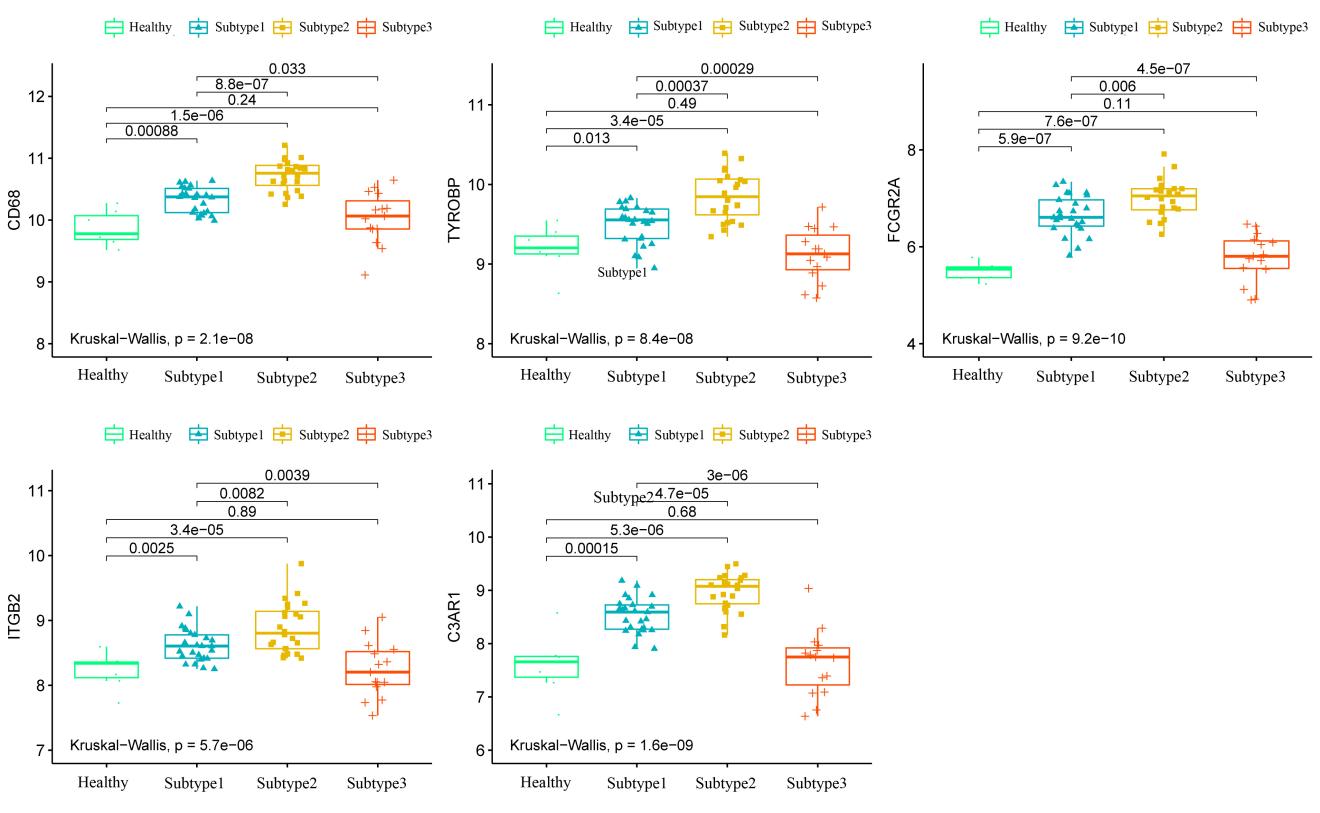


### Supplementary Figure 6. Kyoto Encyclopedia of Genes and Genomes (KEGG) analysis of representative genes of subtype 2. Counts refer to the number of genes enriched into the relevant pathway and the larger the dotplot, the more genes are enriched. Different colors represented different p.values.
